# Disparities in self-reported mental health, physical health, and substance use across sexual orientations in Canada

**DOI:** 10.1371/journal.pone.0305019

**Published:** 2025-03-17

**Authors:** Zachary Bellows, Chungah Kim, Yihong Bai, Peiya Cao, Antony Chum

**Affiliations:** 1 School of Kinesiology and Health Science, York University, Toronto, Ontario, Canada; 2 Department of Epidemiology and Biostatistics, Western University, London, Ontario,; Canada; 3 Dalla Lana School of Public Health, University of Toronto, Toronto, Ontario, Canada; Northern Arizona University, UNITED STATES OF AMERICA

## Abstract

**Background:**

While prior studies have shown LGB individuals have elevated risk of poor mental health, poor physical health, and substance use, existing study designs may be improved by using representative samples, wider ranges of health outcomes, heterosexual comparison groups, and disaggregated data. The goal of this study is to provide estimates of multiple health disparities across sexual orientations in Canada based on these principles.

**Methods:**

Using data from 2009-2014 Canadian Community Health Surveys, a sample of 19,980,000 weighted individuals was created. Outcomes included mental health, physical health, binge drinking, illicit drug use, and cannabis use. The study used logistic regression models adjusted by covariates, stratified by sex, to estimate health disparities across sexual orientations over time.

**Results:**

Among LGB individuals, there was evidence for elevated risk of poor mental health (i.e. gay men, bisexual men, bisexual women), poor physical health (i.e. bisexual men, bisexual women), binge drinking (i.e. lesbians, bisexual women), illicit drug use (i.e. lesbians, bisexual women), and cannabis use (i.e. lesbians, bisexual women) relative to their heterosexual counterparts. Those identifying as ‘don’t know’ or ‘refuse’ showed reduced odds of substance use. Bisexual women exhibited highest disparities in health outcomes, e.g. OR=3.3, 95% 2.58 to 4.22 for poor mental health. Trends over time showed worsening mental health among bisexual women (relative to changes in heterosexual women), and decreasing substance use in gay and bisexual men, and lesbians.

**Conclusion:**

This study highlights health disparities across sexual orientations in Canada, especially bisexual women, calling for targeted interventions (e.g. increased training of service providers in working with bisexual women and community outreach against biphobia). Future research should aim to explore these disparities longitudinally while also including the use of administrative-linked health data to reduce potential bias in self-reported data.

## Introduction

Promoting health equity in marginalised communities is a fundamental objective of public health; however, to address these disparities effectively, it is crucial to understand their magnitude. The purpose of this study is to provide an estimate of health disparities across sexual orientations with regards to mental health, physical health, alcohol consumption, and substance use (illicit drug use and cannabis use). To contextualize our research, we draw on the minority stress model as a theoretical framework, identifying key gaps in prior literature. Finally, we outline our study objectives and research questions, which aim to provide a comprehensive and nuanced understanding of these health disparities across sexual orientations.

### Prior literature and the minority stress model as a theoretical framework

The minority stress model posits that belonging to a minority group inherently presents unique social challenges, leading to elevated stress responses and adverse health outcomes[1,2]. With regards to health disparities across sexual orientation, the model specifically highlights how clashes with heteronormative culture can engender environments where sexual minorities face distinctive stressors such as internalisation of negative societal attitudes, anticipation of rejection, and experiences of discrimination[3]. These stressors subsequently contribute to poor mental health [4,5], physical health conditions [6,7], and may even develop into substance misuse as a coping strategy [8,9]. While the minority stress model has contributed to the theoretical foundations for the study of health disparities across sexual orientations, prior studies are characterised by key limitations that we aim to address in our present study, they include: 1) testing the minority stress theory with a narrow set of health outcomes, 2) limitations of convenience samples, 3) missing heterosexual comparison group, and 4) the lack of appropriate disaggregation across sexual orientation groups.

### Testing the minority stress theory using a narrow set of health outcomes

White et al. argue that traditional explanatory models in epidemiology often focus on specific health conditions by identifying disease-specific risk factors [10]. However, social exposures, such as the impact of discrimination, are likely to have more generalized and cumulative effects on health, which may not be fully captured by studies centered on individual diseases or conditions [11]. The practice of disease-specific epidemiological investigations is also common among studies of sexual minority health, with many prior studies examining disparities across sexual orientation in a narrow range of health outcomes such as substance use [12], mental health [13], and physical health [14]. Yet, the mechanism through which minority stress is theorised to impact health outcomes, namely minority stress [8], are not specific to any given disorder or condition and are expected to have a generalised, cumulative health impact. Therefore, studies that focus narrowly on a single disease/disorder, rather than focusing on domains of health (such as mental health or physical health), have been argued by Frost [15], to risk “false null” findings that could imply social disadvantage does not affect the health of sexual minorities. Therefore, if possible, studies of disparities in health status across sexual orientation would benefit from the examination of a wide range of health outcomes to capture the generalised health impact of minority stress.

### Limitations of convenience samples

A number of studies that examined sexual minority health have relied on convenience samples or small, non-representative samples[13,16–19]. Convenience sampling involves selecting participants based on their availability and ease of recruitment [20], which limits the generalizability of the findings since the characteristics of those who volunteer as participants may systematically differ from non-participants. Among these studies, participants were often recruited at Pride and community events, with postcards advertising the study being distributed LGBTQ+ (i.e. lesbian, gay, bisexual, transgender, queer, and others) community organisations, and was supplemented by advertisement on radio, social media websites, and mobile meet-up apps such as Grindr. These recruitment strategies may lead to a sample who are over-represented by those who are well connected to the LGBTQ+ community, while those who are isolated from the LGBTQ+ community or not out of the closet are less likely to be recruited leading to potential sampling bias [20]. Therefore, if people who are isolated have a systematically different health status compared to those who are well-connected, as suggested by prior literature [21], then prior studies using convenience samples will likely be biased.

### Missing heterosexual comparison group

Some previous studies have investigated sexual minority health without including a heterosexual comparison group [13,16,22–25], which makes it difficult to quantify disparities and measure the impact of minority stress, since comparing health outcomes between sexual minority groups who face minority stress and heterosexual individuals are required to highlight disparity. Using a heterosexual comparison group provides a reference point that allows us to determine the extent of health disparities across sexual orientations. While these studies helped characterise health problems in sexual minority individuals, the use of comparison groups would help contextualise these health problems (i.e. understand the risk irrespective of sexual minority status) and shed light on the specific challenges faced by lesbian, gay, and bisexual (LGB) individuals.

### Issues with no disaggregation across sex or sexual minority groups

Another limitation in the literature is common practice of the aggregating lesbian, gay, and bisexual, individuals into a single “LGB” or sexual minority category in prior studies [26–29]. This approach can be problematic since it may obscure important disparities between these groups. Specifically, bisexual individuals may face unique challenges: 1) marginalisation by both heterosexual and gay/lesbian communities [30], 2) experience of biphobia and invalidation (e.g. being dismissed or excluded based on prejudice and stereotypes about bisexual individuals) [31], and 3) encounter mistrust and stereotypes (e.g. related to their ability to commit) that affect their relationships and overall well being [32]. Disaggregating gay/lesbians from bisexuals may reveal important health disparities that are hidden in the data.

Another related issue is that even when a study disaggregates gays/lesbians from bisexuals, men and women may be aggregated into a single group, and gender/sex is typically used as an adjustment variable in these studies [33–35], which can lead to misestimations since there may be significant gender/sex differences within a given sexual orientation (e.g. gay men vs. lesbians). While disaggregated analysis by sex allows second level sex differences to be revealed across all model covariates [36], it comes at a cost to statistical power that may be too high for many studies except for large scale population-based samples collected by national statistical bureaus.

### Rurality as a potential effect modifier

Although previous research has extensively documented health disparities across sexual orientations, there are limited studies of how these disparities might be modified by rurality status. Living in rural areas may exacerbate the challenges faced by sexual minority individuals, as they often face additional stressors, such as heightened stigma, fewer LGB-specific social networks, and limited access to mental health services tailored to their needs [37]. Despite these unique challenges, few studies have examined the impact of rurality in modifying health disparities across sexual orientations. One exception is a study that investigated whether rurality amplifies the risk of suicide-related behaviours among sexual minority individuals in Ontario. This study found that both sexual minority status and rural residence independently increased the risk of suicide-related behaviours, but no significant interaction was observed between these two factors. This gap in the literature highlights the need for further research to explore whether rurality interacts with sexual orientation to shape broader health outcomes beyond suicide-related behaviours.

Our study objectives are to 1) investigate disparities in mental health, physical health, binge drinking, illicit drug use, and cannabis use across sexual orientations; and 2) explore trends in these disparities over time between 2009 and 2014. This research makes several unique contributions to the literature. First, by examining a broad range of health outcomes, we move beyond the disease-specific approaches often employed in prior studies, providing a comprehensive assessment of the cumulative health impact of minority stress across multiple domains. Second, we utilize a nationally representative dataset, enabling us to capture the experiences of sexual minorities in remote areas and those who may be disconnected from LGBTQ+ communities, which are often underrepresented in convenience samples. Third, we include a heterosexual comparison group, providing a critical reference point for assessing the extent of health disparities and the impact of minority stress. Finally, our study disaggregates data by both sexual orientation and sex, uncovering nuanced differences within the sexual minority population that are often obscured by aggregation. These contributions enhance our understanding of the diverse health challenges faced by sexual minorities and offer valuable insights for addressing these disparities. We examined changes between 2009 to 2014 to investigate whether the health disparities between sexual orientations changed over time, and whether the health disparities across sexual orientations are further modified by rurality. Our research questions are as follows:

1) What are the disparities in mental health, physical health, binge drinking, illicit drug use, and cannabis use across sexual orientations (lesbian, gay, bisexual, and heterosexual individuals) in Canada, and how do these disparities vary between men and women?
2) How have these health disparities across sexual orientations changed over time, specifically between 2009 and 2014, and do they reflect an improvement or worsening in health disparities?
3) Does rurality modify the association between sexual orientation and health disparities?

## Methods

### Sample

The Canadian Community Health Survey (CCHS) is a cross-sectional survey conducted annually across Canada, utilizing multistage sampling and computer-assisted telephone interviews in English or French. This study includes data from six cycles (2009-2014), comprising a weighted sample of 19,980,000 individuals aged 18-59. Survey weights were used to ensure the representativeness of the Canadian population. The response rates of the six cycles ranged from 87.3% [38] to 89.3% [39].

Secondary data analysis was conducted without obtaining written informed consent from participants for this research. This study adhered to the data confidentiality guidelines set by Statistics Canada and the Statistics Canada Research Data Centre. The data were anonymized, and the research team did not have access to the personal identifiers of CCHS participants. The study has received ethical approval from the York University Research Ethics Board (certificate number: 20-134- CHUM).

### Exposure variable

Participants were asked to indicate their sex as either male or female. In order to determine sexual orientation, participants were asked, “Do you consider yourself to be…”, and were given the following response options: heterosexual, homosexual, bisexual, or ‘don’t know’, or ‘refuse to say’. When compared to questionnaires that encompass sexual identity, behaviour, and attraction through a multi-question approach, this single-question approach has been shown to be a reliable measure and has displayed a strong correlation with sexual identity (kappa statistic of 0.89) [40]. In previous versions of the CCHS, the single-question method successfully identified 99.3% of participants who identified as a sexual minority based on a comprehensive questionnaire, as well as 84.2% of those who reported engaging in same-sex relationships at any point in their lives [40]. In this study, sexual orientation is represented by five categories: heterosexual, gay/lesbian, bisexual, ‘don’t know’, and ‘refuse to say’.

### Outcome variables

Outcomes included mental and physical health, binge drinking, illicit drug use, and cannabis use. Mental health and physical health were dichotomized as: excellent, very good, good, vs. fair, poor for physical and mental health. A scoping review on the validity of single item self-rated mental health found that ratings of fair and poor self-rated mental health had 4.57 to 9.97 times higher risk of being diagnosed with major depressive disorder [41]. Similarly, fair and poor physical self-rated health has been associated with a 2-fold higher mortality risk compared with persons reporting a higher health status [42].

Binge drinking was determined by any instance of self-reported binge drinking in the past 12 months, where binge drinking is defined by the CCHS as a male having more than four standard alcoholic drinks in one occasion or a female having more than three standard alcoholic drinks in one occasion. Illicit drug use was determined by a derived drug use variable based on questions asking the participant if they used the following drugs in the past 12 months: cocaine or crack, speed/amphetamines, ecstasy/MDMA, hallucinogens, PCP (Phencyclidine), LSD/acid (Lysergic acid diethylamide), sniffing glue, gasoline, other solvents, and heroin.

Cannabis use was determined by its use in the past 12 months, based on a self-reported response to essentially: “During the past 12 months have you used marijuana?”. Previous studies have provided validation for self-administered single-item screening questions aimed at detecting unhealthy substance use. These studies indicate that individuals with substance use conditions are at least three times more likely to yield a positive screen and are less than one-third as likely to yield a negative screen [43]. Furthermore, the reliability of self-rated substance consumption measures has been demonstrated in prior literature. For instance, the reliability coefficient (kappa) for binge drinking alcohol stands at 0.76, while it ranges from 0.72 (for hallucinogens) to 0.76 (for cocaine) for illicit drug use. Additionally, the reliability coefficient for cannabis use is reported at 0.82 [44]. These findings underscore the utility and validity of self-administered screening questions and self-rated substance consumption measures in identifying and assessing unhealthy substance use behaviours.

### Covariates

The following variables were used in the models as statistical controls: 1) year of birth (continuous), 2) marital status (married or common law vs. single, separated, divorced, or widowed), 3) educational attainment (achieved at least a post-secondary degree/certificate vs. not), 4) student status (current student vs. not), 5) self-reported ethnic minority status (yes vs. no), 6) employment status (Any work in the last year vs. not), 7) rurality status (yes vs. no) based on prescribed rural postal codes [45], 8) province of residence, 9) year of interview as dummies (2009-2014), and 10) income.

### Statistical analysis

First, cross-tabulations of sexual orientations and outcomes, along with covariates, are calculated. Second, logistic regression models were used to estimate differences in outcomes across sexual orientations. All models were stratified by sex. Unadjusted models and adjusted models were estimated for each outcome. Multiple imputation by chained equations was used to impute missing values in all covariates.

The following sensitivity tests were also performed: 1) models using a modified Poisson regression; 2) models using complete case analyses (dropping cases with missing data instead of multiple imputations); 3) models with interactions between sexual orientation and year of interview to check for potential change over the study period; and 4) models with interactions between sexual orientation and rurality was tested by calculating and testing the predicted probabilities. All analysis was conducted using STATA v17.

## Results:

Table 1 shows the descriptive statistics of the study participants (weighted n= 19,980,000), which includes cross-tabulations between sexual orientation with each outcome and sociodemographic covariates (e.g. age, income, ethnicity, etc.). Bisexual respondents reported the highest rates of poor physical (17.67%), mental health (20.47%), binge drinking (61.4%), illicit drug use (13.02%), and cannabis use (13.02%). Bisexuals and ‘don’t know’ respondents tended to occupy lower income quintiles, with bisexuals notably concentrated in the lowest quintile (30.23%). Gay/lesbian and heterosexual individuals appeared more frequently in higher income quintiles. Gay/lesbian and bisexual groups were less likely to be married and live in rural areas. Employment rates were highest among heterosexual and gay/lesbian respondents.

**Table 1:** Descriptive statistics by sexual orientation: mental and physical health, substance use, demographics, and socioeconomic indicators from the canadian community health survey.

	Heterosexual	Gay/lesbian	Bisexual	Don’t Know	Refuse	Total
**Number of Persons**	19,122,000	284,000	215,000	59,000	120,000	19,980,000
**Poor Physical Health, N** **(column %)**	1,623,000 (8.49%)	28,000 (9.86%)	38,000 (17.67%)	8,000 (13.56%)	18,000 (15.00%)	1,767,000 (8.84%)
**Poor Mental Health, N (column %)**	1,121,000 (5.86%)	26,000 (9.15%)	44,000 (20.47%)	6,000 (10.17%)	13,000 (10.83%)	1,210,000 (6.06%)
**Binge Drank Alcohol** ^**1**^ **, mean (column %)**	9,527,000 (49.82%)	169,000 (59.51%)	132,000 (61.40%)	11,000 (18.64%)	29,000 (24.17%)	9,906,000 (49.58%)
**Illicit Drug Use** ^**2**^ **, N** **(column %)**	962,000 (5.03%)	22,000 (7.75%)	28,000 (13.02%)	1,000 (1.69%)	3,000 (2.50%)	1,016,000 (5.09%)
**Cannabis Use** ^**3**^ **, N** **(column %)**	987,000 (5.16%)	22,000 (7.75%)	28,000 (13.02%)	1,000 (1.69%)	3,000 (2.50%)	1,041,000 (5.21%)
**Mean Age (standard deviation)**	39 (12.11)	39 (12.36)	32 (11.96)	41 (12.41)	40 (12.39)	39 (12.15)
**Married, N** **(column %)**	11,948,000 (62.48%)	102,000 (35.92%)	64,000 (29.77%)	31,000 (52.54%)	58,000 (48.33%)	12,280,000 (61.46%)
**Has Completed Post-Secondary, N** **(column %)**	12,328,000 (64.47%)	208,000 (73.24%)	104,000 (48.37%)	26,000 (44.07%)	66,000 (55.00%)	12,783,000 (63.98%)
**Current Student, N** **(column %)**	2,577,000 (13.48%)	46,000 (16.20%)	55,000 (25.58%)	6,000 (10.17%)	17,000 (14.17%)	2,727,000 (13.65%)
**White, N** **(column %)**	14,460,000 (75.62%)	240,000 (84.51%)	164,000 (76.23%)	30,000 (50.85%)	62,000 (51.67%)	15,082,000 (75.49%)
**Employed in the last year, N** **(column %)**	16,767,000 (87.68%)	255,000 (89.79%)	173,000 (80.47%)	39,000 (66.10%)	90,000 (75.00%)	17,324,000 (86.71%)
**Lives Rural, N** **(column %)**	2,898,000 (15.16%)	26,000 (9.15%)	22,000 (10.23%)	8,000 (13.56%)	11,000 (9.17%)	2,994,000 (14.98%)
**Income Quintile**						
Q1 (Lowest), N (column %)	3,082,000 (16.11%)	42,000 (14.79%)	65,000 (30.23%)	21,000 (35.59%)	35,000 (29.17%)	3,291,000 (16.47%)
Q2, N (column %)	3,211,000 (16.79%)	45,000 (15.85%)	41,000 (19.07%)	9,000 (15.25%)	20,000 (16.67%)	3,366,000 (16.85%)
Q3, N (column %)	3,627,000 (18.97%)	47,000 (16.55%)	47,000 (21.86%)	10,000 (16.95%)	15,000 (12.50%)	3,778,000 (18.91%)
Q4, N (column %)	4,049,000 (21.17%)	57,000 (20.07%)	29,000 (13.49%)	7,000 (11.86%)	14,000 (11.67%)	4,184,000 (20.94%)
Q5 (Highest), N (column %)	4,310,000 (22.54%)	84,000 (29.58%)	23,000 (10.70%)	5,000 (8.47%)	8,000 (6.67%)	4,452,000 (22.28%)

^1^ **Defined as 4 or more standard drinks for females during one occasion in the past 12 months, and 5 or more standard drinks for males during one occasion in the past 12 months.**

^2^ **Defined as any use in the past 12 months.**

^3^ **Defined as any use in the past 12 months.**

^Note^ **Data pooled from 2009 to 2014, total weighted sample size n=19,980,000 individuals.**

Table 2 shows the adjusted (i.e., models 1a–5a) and unadjusted (i.e., models 1b–5b) probabilities of reporting poor mental health, poor physical health, binge drinking, illicit drug use, and cannabis use among men. In the fully adjusted models, bisexual men had the highest probabilities of reporting poor mental health (12.0%), illicit drug use (9.2%), and cannabis use (8.6%). Gay men followed, with predicted probabilities of 8.1% for poor mental health, 6.7% for illicit drug use, and 7.0% for cannabis use. Heterosexual men ranked third highest in these outcomes. For binge drinking, heterosexual men had the highest probability (59.4%), followed by bisexual men (58.5%) and gay men (57%). Regarding poor physical health, those who refused to answer had the highest probability of reporting poor physical health (11.5%), followed by bisexual men (11.5%) and gay men (9.5%).

**Table 2:** Male adjusted and unadjusted odds ratios for poor mental health, poor physical health, binge drinking, using illicit drugs, and using cannabis across sex and sexual orientation for Canadians.

Sexual Orientations	Predicted Probability		95% CI		Predicted Probability		95% CI	
		Adjusted models				Unadjusted models		
	Model 1a: Poor Mental Health (Male)				Model 1b: Poor Mental Health (Male)			
Heterosexual	5.2%		4.9%	5.5%	5.4%		5.1%	5.7%
Gay men	8.1%		5.8%	10.4%	9.3%		7.0%	8.6%
Bisexual	12.0%		8.6%	15.4%	19.2%		14.1%	24.2%
Don’t Know	4.9%		1.4%	8.5%	9.4%		4.5%	14.3%
Refuse	7.4%		0.9%	13.9%	10.4%		4.0%	16.9%
	Model 2a: Poor Physical Health (Male)				Model 2b: Poor Physical Health (Male)			
Heterosexual	8.0%		7.6%	8.3%	8.2%		7.9%	8.6%
Gay men	9.5%		7.0%	12.0%	9.9%		7.3%	12.4%
Bisexual	11.5%		8.0%	15.1%	16.4%		11.6%	21.1%
Don’t Know	5.5%		2.2%	8.8%	11.0%		6.0%	16.0%
Refuse	12.2%		4.7%	19.6%	14.9%		7.9%	22.0%
	Model 3a: Binge Drinks Alcohol (Male)				Model 3b: Binge Drinks Alcohol (Male)			
Heterosexual	59.4%		58.8%	60.1%	59.6%		58.9%	60.3%
Gay men	57.0%		52.5%	61.4%	61.8%		57.3%	66.4%
Bisexual	58.5%		50.6%	66.5%	59.5%		52.6%	66.3%
Don’t Know	31.4%		20.0%	42.9%	27.4%		17.2%	37.7%
Refuse	37.6%		26.3%	48.8%	32.3%		23.6%	41.0%
	Model 4a: Uses Illicit Drugs (Male)				Model 4b: Uses Illicit Drugs (Male)			
Heterosexual	6.2%		5.9%	6.5%	6.5%		6.2%	6.9%
Gay men	6.7%		4.5%	9.0%	8.4%		5.8%	11.0%
Bisexual	9.2%		5.6%	12.9%	11.3%		7.4%	15.2%
Don’t Know	0.9%		0.1%	1.7%	1.2%		0.4%	1.9%
Refuse	2.3%		0.4%	4.3%	3.2%		1.1%	5.3%
	Model 5a: Uses Cannabis (Male)				Model 5b: Uses Cannabis (Male)			
Heterosexual	6.3%		6.0%	6.6%	6.7%		6.3%	7.0%
Gaymen	7.0%		4.5%	9.5%	8.3%		5.6%	11.0%
Bisexual	8.6%		5.3%	12.0%	10.8%		7.1%	14.4%
Don’t Know	0.9%		0.1%	1.7%	1.2%		0.4%	1.9%
Refuse	2.0%		0.2%	3.8%	3.0%		0.9%	5.0%

^Note^ **Data pooled from 2009 to 2014, total weighted sample size n=19,980,000 individuals. Adjusted models are controlled for variables including year of birth, marital status, educational attainment, student status, self-reported ethnic minority status, employment status, rurality status, province of residence, year of interview, and federal income.**

Table 3 showed the results for women. In our fully adjusted models, similar patterns emerged. Bisexual women reported the highest probability of reporting poor mental health (17.6%), binge drinking (50.2%), using illicit drugs (7.1%), and using cannabis (7.4%). They were followed by lesbians, with a predicted probability of 7.9% reporting poor mental health, 5.2% reporting using illicit drugs, and 5.2% for using cannabis. For binge drinking, bisexual women reported the highest probability (50.2%), followed by lesbians (49.1%), and heterosexual women (40.3%). In terms of poor physical health, bisexual women reported the highest probability (17.4%), followed by those who refused to answer (10.7%), and lesbian women (9.8%).

**Table 3:** Female adjusted and unadjusted odds ratios for poor physical health, poor mental health, binge drinking, using illicit drugs, and using cannabis across sex and sexual orientation for Canadians.

Sexual Orientations	Predicted Probability	95% CI		Predicted Probability	95% CI	
	Adjusted models			Unadjusted models		
	Model 1a: Poor Mental Health (Female)			Model 1b: Poor Mental Health (Female)		
Heterosexual	6.2%	5.8%	6.5%	6.3%	6.0%	6.7%
Lesbian women	7.9%	5.6%	10.2%	8.6%	6.2%	11.0%
Bisexual	17.6%	14.0%	21.2%	21.5%	17.4%	25.5%
Don’t Know	7.3%	3.0%	11.6%	10.4%	5.2%	15.6%
Refuse	5.7%	1.9%	9.5%	11.7%	5.8%	17.6%
	Model 2a: Poor Physical Health (Female)			Model 2b: Poor Physical Health (Female)		
Heterosexual	8.4%	8.0%	8.7%	8.7%	8.4%	9.1%
Lesbian women	9.8%	7.0%	12.6%	9.9%	7.1%	12.6%
Bisexual	17.4%	13.7%	21.1%	18.3%	14.5%	22.1%
Don’t Know	6.1%	2.6%	9.7%	14.8%	7.6%	22.0%
Refuse	10.7%	3.4%	18.0%	15.2%	8.9%	21.6%
	Model 3a: Binge Drinks Alcohol (Female)			Model 3b: Binge Drinks Alcohol (Female)		
Heterosexual	40.3%	39.7%	40.9%	40.4%	39.8%	41.1%
Lesbian women	49.1%	43.1%	55.0%	56.6%	50.7%	62.5%
Bisexual	50.2%	45.4%	55.0%	63.1%	58.8%	67.5%
Don’t Know	20.5%	13.6%	27.3%	14.2%	8.4%	19.9%
Refuse	29.6%	15.0%	44.2%	19.7%	11.7%	27.7%
	Model 4a: Uses Illicit Drugs (Female)			Model 4b: Uses Illicit Drugs (Female)		
Heterosexual	3.3%	3.1%	3.6%	3.5%	3.3%	3.8%
Lesbian women	5.2%	3.6%	6.9%	6.9%	4.8%	9.1%
Bisexual	7.1%	5.0%	9.2%	13.7%	10.6%	16.8%
Don’t Know	1.0%	-0.7%	2.7%	1.0%	-0.2%	2.3%
Refuse	1.8%	-0.3%	3.9%	2.2%	-0.3%	4.8%
	Model 5a: Uses Cannabis (Female)			Model 5b: Uses Cannabis (Female)		
Heterosexual	3.4%	3.2%	3.6%	3.7%	3.4%	3.9%
Lesbian women	5.2%	3.5%	6.9%	6.9%	4.7%	9.0%
Bisexual	7.4%	5.3%	9.5%	14.0%	10.8%	17.1%
Don’t Know	1.4%	-0.4%	3.3%	1.3%	-0.1%	2.6%
Refuse	1.7%	-0.4%	3.8%	2.3%	-0.3%	4.8%

^Note^ **Data pooled from 2009 to 2014, total weighted sample size n=19,980,000 individuals. Adjusted models are controlled for variables including year of birth, marital status, educational attainment, student status, self-reported ethnic minority status, employment status, rurality status, province of residence, year of interview, and federal income.**

The 4 sensitivity test results are overall consistent with the findings from main models. To begin with, the modified Poisson regression analyses (see S3 Table models 1c-5c for males and S4 Table models 6c-10c for females) confirmed these patterns of significance, showing consistent differences for the same groups when compared to their heterosexual counterparts. Second, models using complete case analyses (S3 Table models 1d-5d for male, S4 Table models 6d-10d for female) showed similar directionality, strength of association, and patterns of significant results compared to the imputed main analyses. Weighted number of item non-response for all variables can be found in S2 Table (weighted n=19,980,000), where level of missingness varied from 0% for variables such as student status and rurality to the highest at 4.49% for reports of income quintile. The third sensitivity test showed the trends of disparities (between each sexual orientation group vs. heterosexual) over time (S1-S10 Figs), based on the sexual orientation interaction with ‘year of interview’ are shown in the linear trends are tested using the Mann-Kendall Trend Test (S1 Table). Bisexual women were the only group to show evidence of an increasing disparity in mental health over our study period from 2009-2014. Evidence for a decreasing disparity in substance use and cannabis use was found between bisexual men and heterosexual men.

S5 Table shows the second difference test to estimate the health disparities between each sexual minority group (lesbian/gay, bisexual, don’t know/refused) vs heterosexual individuals in urban vs. rural settings. Our results indicate that there is no evidence that the health disparities across sexual orientation is different in urban vs rural settings.

## Discussion

Our study provides details of health disparities in a broad set of health outcomes across sexual orientation using a population-representative sample of Canada. Among LGB participants, there were evidence for elevated risk of poor mental health (i.e. gay men, bisexual men, bisexual women), poor physical health (i.e. bisexual men, bisexual women), binge drinking (i.e. lesbians, bisexual women), illicit drug use (i.e. lesbians, bisexual women), and cannabis use (i.e. lesbians, bisexual women) relative to their heterosexual counterparts. While prior studies have generally highlighted worse health outcomes among sexual minorities, our study provides evidence that the level and number of health disparities vary among sexual minority groups: from gay men showing elevated risk in 1 outcome tested (i.e. poor mental health) to bisexual women who had elevated risk across all 5 outcomes tested. Furthermore, the strength of disparity varies widely between groups, e.g. with lesbians having 76% increased odds of cannabis use relative to heterosexual women, while bisexual women have 133% increased odds. Over the study period from 2009 to 2014, we observed worsening mental health among bisexual women (relative to heterosexual women) and improved substance use outcomes among gay men, bisexual men, and lesbian women (relative to their heterosexual counterparts). In addition, our results indicate that health disparities across sexual orientation do not differ between urban and rural settings.

### Substance use in sexual minority women

A previous study examining the association between sexual orientation and substance use hospitalisation across Canada (also based on the Canadian community health survey) found that while bisexual women had elevated odds of substance use hospitalizations by a factor of 2.46 compared to their heterosexual counterpart [46], no elevated risk was detected in other sexual minority groups. Given that bisexual women had elevated risk in each substance use outcome in our study, and had the largest disparities relative to their heterosexual counterparts (among all other LGB groups), it was not surprising to see that this group also had heightened risk for substance-related hospitalisation. On the other hand, lesbians in our study also exhibited increased risk for all 3 substance use outcomes (with a smaller effect size compared to bisexual women); however, there was no evidence in the prior study that the lesbian group had higher risk of hospitalizations for any substances (Hazard Ratio, HR=0.98, 95% CI 0.47 to 2.06), alcohol-related hospitalizations (HR=0.98, 95% CI 0.34 to 2.86), or illicit drugs and cannabis hospitalizations (HR=0.96, 95% 0.38 to 2.39). While both lesbians and bisexual women report high levels of substance use in our study, only bisexual women have a higher risk of hospitalization. This discrepancy is aligned with findings from a review indicating that bisexual identity is often associated with more problematic substance use than those identifying as strictly heterosexual or gay/lesbian respondents [47]. This literature helps to explain why, among women, despite elevated risks of substance use in lesbians and bisexual women, only the bisexual group was linked to increased substance use hospitalizations.

### Substance use in ‘don’t know’ and ‘refuse’ individuals

While all participants in the ‘refused’ and ‘don’t know’ had even odds of poor physical and mental health relative to their heterosexual counterparts, many reported significantly reduced odds of 3 substance use (i.e. binge drinking, illicit drugs, and cannabis) outcomes. These results starkly contrast with a prior study that examined substance-related hospitalisation across sexual orientation [46]. More specifically, it showed that the ‘don’t know’ and ‘refused’ groups (combined into a single ‘other’ group) had elevated risk of age-adjusted incidence rates for substance-use hospitalizations [46]. In fully adjusted models, no differences were shown between the ‘other’ groups and their gender matched heterosexual counterparts for all substances examined. The discrepancy between self-reported substance use and substance use-related hospitalisation rates among the ‘don’t know’ and ‘refused’ groups raises questions about the accuracy of self-reports in these groups. Further research should investigate whether social desirability bias or fear of stigma associated with substance use may affect self-reported data in groups that are also reluctant to disclose their sexual orientations.

### Mental and physical health in sexual minority individuals

Among LGB men and women, all groups, except for lesbians, showed elevated odds of reporting poor mental health. A prior literature review has shown that, in most studies, LGB individuals report higher risk of mental health problems [48]. However, in a controlled sibling study, involving comparison of lesbians to their heterosexual sisters found no statistically significant differences in mental health, and even found lesbians to have higher rates of self esteem [49]. The author hypothesised that for lesbians, their degree of disclosure about their sexual orientation, or ‘outness’, may have acted as a protective factor in their study participants. Our study, based on self-reported mental health, also provides some evidence that lesbians may uniquely be protected against poor mental health among sexual minority groups.

Our study shows that while gay and lesbian individuals had similar risk of poor physical health as their heterosexual counterparts, bisexual men and women had higher risk of poor physical health. Prior studies on differences in physical health across sexual orientations had different results, which may be due to differences in how gender and sexual orientation are disaggregated. For example, a recent study using the US General Social Survey [50], found that after controlling for sociodemographic variables, no LGB groups have significant differences in their physical health over the past 30 days relative to their heterosexual counterparts; however, the study did not have gender disaggregated analyses, or used sex-interactions, to explore sex/gender effect modification (leaving the possibility that the disparity between bisexual men vs gay men may be different than bisexual women vs lesbian). In a Swedish nationally representative study [7], after statistical adjustments, LGB individuals reported higher odds of physical symptoms (e.g., pain, insomnia, dermatitis, tinnitus, intestinal problems) and conditions (e.g., diabetes, asthma, high blood pressure) compared to heterosexuals; however, the study did not disaggregate gay/lesbian and bisexual participants, and studied sexual minority as a single group, which provided limited insights into the bisexual vs gay/lesbian differences that we found.

Our finding that bisexual individuals (and not gay men/lesbians) had elevated risk of poor physical health may be explained by increased minority stress as past studies have indicated that biphobia is pervasive in heterosexual and LGBTQ+ communities [31,51], and as a result, bisexual individuals receive reduced community support and face a higher level of discrimination compared to gay/lesbian individuals [52]. In a systematic review [6], minority stress has been linked to poor physical health through direct physiological stress response (e.g. immune dysregulation and allostatic load) and can modify health behaviours through distress/psychopathology. Given the elevated levels of minority stress in bisexual individuals, it is unsurprising that both mental and physical health disparities are heightented in bisexual individuals.

The analysis of time trends has shed light on the changing dynamics of health outcomes over time and the disparities faced by various sexual orientation groups. These trends highlight the evolving nature of health disparities, though it is important to consider the potential limitations in interpreting these changes due to shifts in population composition across multiple cross-sectional periods. As such, these findings on temporal changes warrant careful consideration. Key observations include: 1) An increasing disparity in mental health issues among bisexual women, which raises concerns given their already higher vulnerability to adverse health outcomes; 2) A significant decrease in substance use disparities among several sexual orientation groups, notably gay and bisexual men for illicit drug and cannabis use, and lesbians for illicit drug use. This decline in substance use among these groups is encouraging. However, the potential widening gap in mental health disparities among bisexual women calls for urgent, focused intervention strategies.

Our study found no significant difference in health disparities across sexual orientation between urban and rural settings. This finding challenges the assumption that rurality exacerbates the health challenges encountered by sexual minorities due to greater stigma, isolation, or reduced access to LGBTQ+ services. While this result may suggest that the stressors related to sexual minority status are pervasive across both rural and urban environments, it may also point to the success of broader, nationwide policies in mitigating geographic differences in health outcomes. Nevertheless, it is critical to continue advocating for LGBTQ+ health services in rural areas to ensure that sexual minorities in these regions have equitable access to care, especially considering the persistent social challenges they may still face.

### Limitations and strengths

There are a number of limitations in our study. First, sexual orientation were based on self-identification, but prior research has shown that a more comprehensive representation should include measures of attraction and sexual behaviour, with evidence showing that certain groups, including heterosexually identified men who have sex with men [53], may have even worse health outcomes compared to gay and bisexual identified men. Second, the study covered multiple years, and the levels of disparity across groups may have changed over these years. To help partially mitigate this concern, sensitivity tests were conducted to investigate the interaction between sexual orientation and time; however, since the data is cross-sectional (and not longitudinal), the observed changes may be driven by compositional change rather than reflecting a larger societal shift. Third, some groups might not openly share their substance use problems, casting doubt on the trustworthiness of their self-reports. Future studies should explore how social pressure or fear of stigma related to substance use could impact self-reported data in these groups, such as those who did not reveal their sexual orientations. Fourth, self-reported health measures may over- or underestimate true health status due to issues like social desirability bias, recall bias, and the inability of participants to accurately quantify their health experiences [54]. Fifth, the study utilized data from 2009-2014, and it is possible that the levels of disparity across groups have evolved since then. While this timeframe may limit the current applicability of our findings, the analyses remain valuable by providing a historical baseline against which to measure future shifts in health disparities. Finally, while the control variables were chosen based on prior literature, there may still be uncontrolled confounders leading to residual confounding.

Despite these limitations, our study has several notable strengths. First, we used a range of health outcomes to capture multiple dimensions of health inequities. By examining various self-rated health outcomes, ranging from physical and mental health to substance use, the study provides a more comprehensive picture of health disparities across sexual orientations in Canada to help inform health promotion strategies. Second, the use of a nationally representative sample is a strength of this study. This was supplemented by the use of population weights provided by Statistics Canada to adjust for potential discrepancies in response rates compared to characteristics found in the Canadian Census, which may improve the generalizability of our study to the Canadian population. Third, the use of disaggregated data for sex and sexual orientation helped to identify significant differences between sexual minority groups (i.e. increased risk of all health outcomes shown in bisexual women), which helps to highlight specific sexual minority subgroups for targeted interventions.

## Conclusions

This study highlights significant health disparities among sexual minorities in Canada, particularly emphasising the unique challenges faced by bisexual women. The findings suggest an urgent need for tailored interventions, as bisexual women not only report higher levels of substance use but also face greater health risks, including hospitalization. A key concern highlighted is the persistence of biphobia and the need for interventions to specifically address this, such as making resources on biphobia readily available in clinical and community settings and ensuring frontline workers are equipped to handle crises related to bisexual stigma. The study also underlines the need for cautious interpretation of self-reported data, especially from individuals uncertain or uncomfortable with disclosing their sexual orientation. Discrepancies noted in self-reported substance use versus administrative data underscore the necessity for further research in clinical settings to ensure accurate health assessments and interventions. Our repeated cross-sectional analysis exposes persistent and sometimes worsening disparities across sexual orientations over time, underscoring the need for future research to adopt longitudinal approaches. Such research would offer insights into evolving health challenges and the effectiveness of past interventions, helping to shape targeted prevention strategies.

In conclusion, this research, utilising a nationally representative sample and comprehensive analyses, provides insights into the complex landscape of health inequities faced by the LGB community. The heightened vulnerability of bisexual women across all assessed health metrics calls for immediate and specific public health responses. Future efforts must continue to refine these interventions and assess their impact longitudinally to ensure they are culturally appropriate and truly effective.

## Supporting information

Fig S1 Odds ratio disparity over time for poor mental health outcome for males 2009-2014. (PDF)

Fig S2 Odds ratio disparity over time for poor mental health for females 2009-2014. (PDF)

Fig S3 Odds ratio disparity over time for poor physical health for males 2009-2014. (PDF)

Fig S4 Odds ratio disparity over time for poor physical health for females 2009-2014. (PDF)

Fig S5 Odds ratio disparity over time for binge drinking for males 2009-2014. (PDF)

Fig S6 Odds ratio disparity over time for binge drinking for females 2009-2014. (PDF)

Fig S7 Odds ratio disparity over time for illicit drug use for males 2009-2014. (PDF)

Fig S8 Odds ratio disparity over time for illicit drug use for females 2009-2014. (PDF)

Fig S9 Odds ratio disparity over time for cannabis use for males 2009-2014. (PDF)

Fig S10 Odds ratio disparity over time for cannabis use for females 2009-2014. (PDF)

Table S1 Results for Mann-Kendall Trend Test across poor mental health, poor physical health, binge drinks, illicit drug use, and cannabis use across sex and sexual orientation for Canadians from 2009-2014. (PDF)

Table S2 Weighted number of item non-response (including don’t know and refused) for all variables. (PDF)

Table S3 Male poisson risk ratios and unimputed odds ratios for poor physical health, poor mental health, binge drinking, using illicit drugs, and using cannabis across sex and sexual orientation for Canadians. (PDF)

Table S4 Female poisson risk ratios and unimputed odds ratios for poor physical health, poor mental health, binge drinking, using illicit drugs, and using cannabis across sex and sexual orientation for Canadians. (PDF)

Table S5 Second-difference test to estimate the difference of (1) - (2): (1) difference between urban sexual minority groups vs urban heterosexual individuals and (2) difference between rural sexual minority groups vs rural heterosexual individuals. Sexual minority groups include gay/lesbian, bisexual, and Don’t know/refuse. (PDF)
