# Supplementary figures and images for: Disparities in self-reported mental health, physical health, and substance use across sexual orientations in Canada
Source: PLoS One. 2025 Mar 17;20(3):e0305019. doi: 10.1371/journal.pone.0305019 (PMC11913302; doi:10.1371/journal.pone.0305019)

**Figure S1. Odds ratio disparity over time for poor mental health outcome for males 2009-2014**

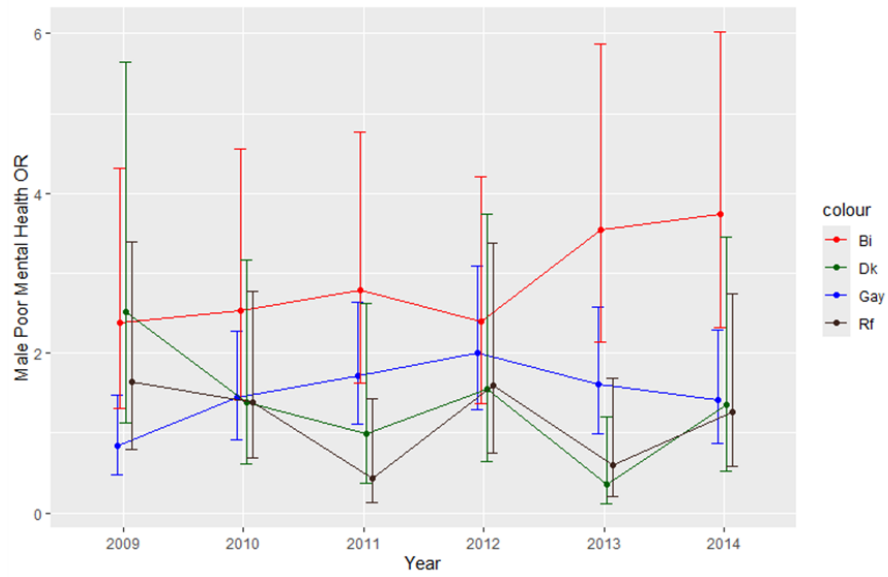

Supplement: Fig S1 — (PDF) [file pone.0305019.s001.pdf]

**Figure S2. Odds ratio disparity over time for poor mental health for females 2009-2014**

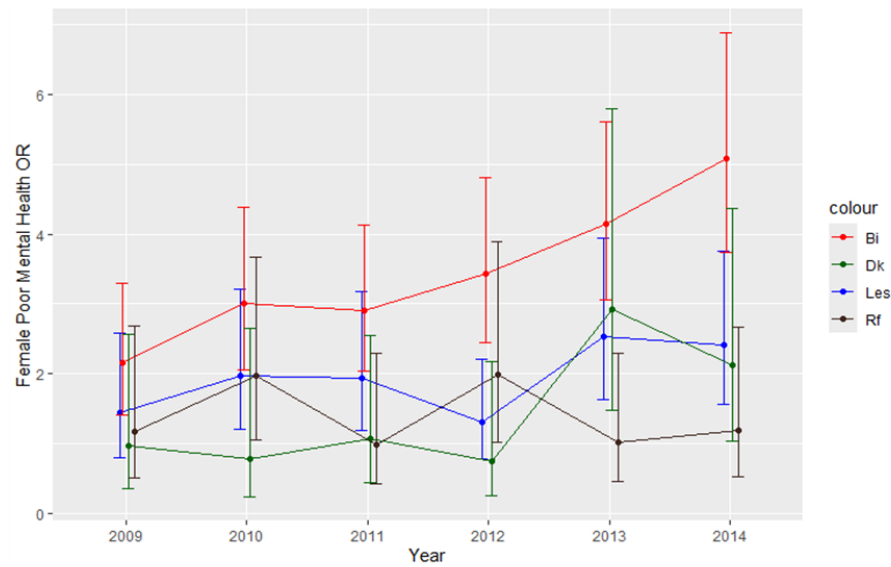

Supplement: Fig S2 — (PDF) [file pone.0305019.s002.pdf]

**Figure S3. Odds ratio disparity over time for poor physical health for males 2009-2014**

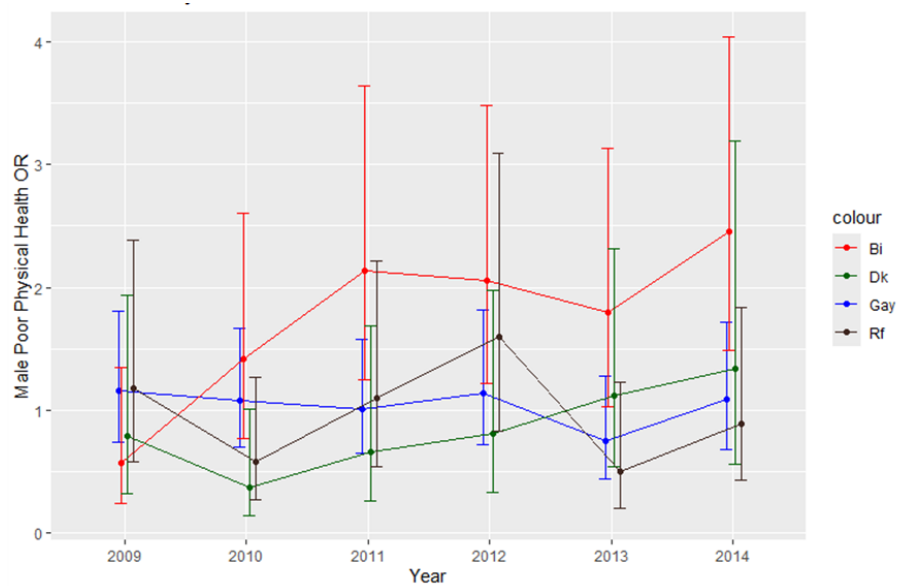

Supplement: Fig S3 — (PDF) [file pone.0305019.s003.pdf]

**Figure S4. Odds ratio disparity over time for poor physical health for females 2009-2014**

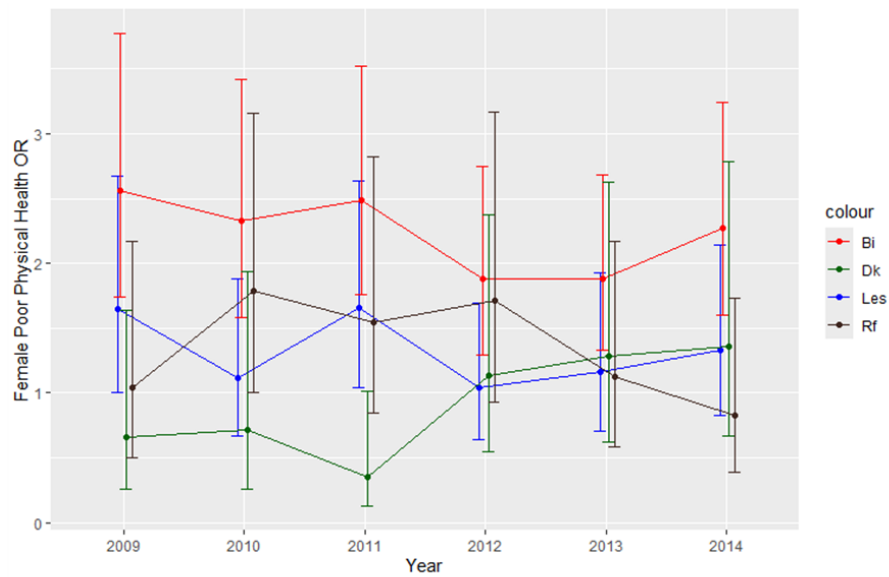

Supplement: Fig S4 — (PDF) [file pone.0305019.s004.pdf]

**Figure S5. Odds ratio disparity over time for binge drinking for males 2009-2014**

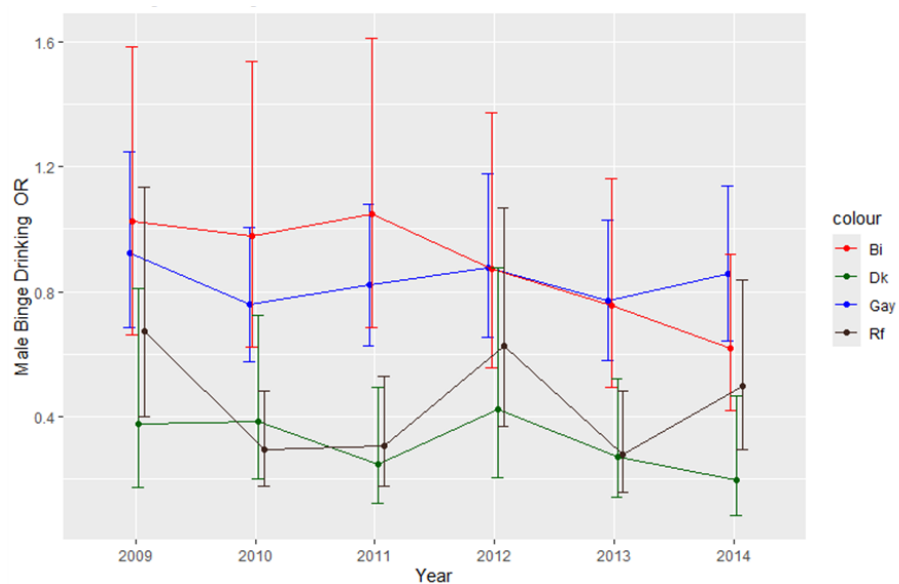

Supplement: Fig S5 — (PDF) [file pone.0305019.s005.pdf]

**Figure S6. Odds ratio disparity over time for binge drinking for females 2009-2014**

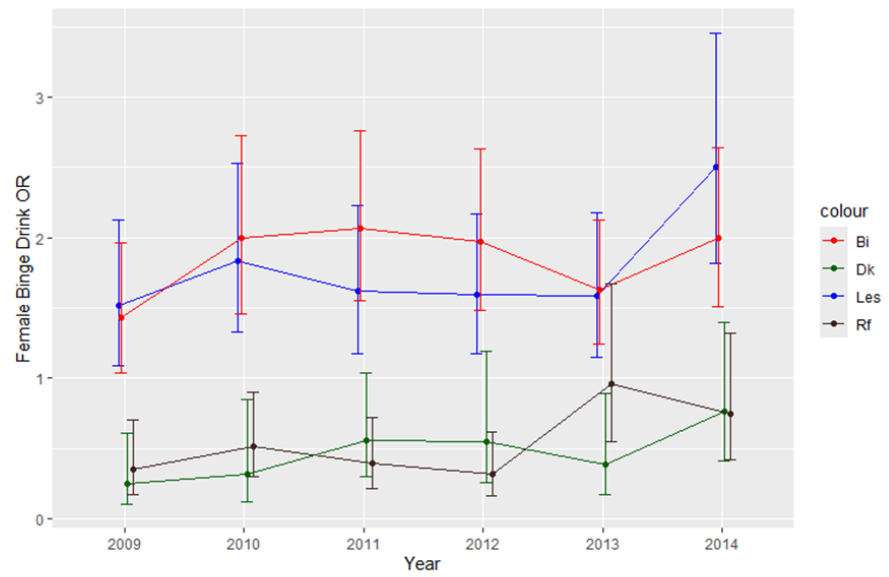

Supplement: Fig S6 — (PDF) [file pone.0305019.s006.pdf]

**Figure S7. Odds ratio disparity over time for illicit drug use for males 2009-2014**

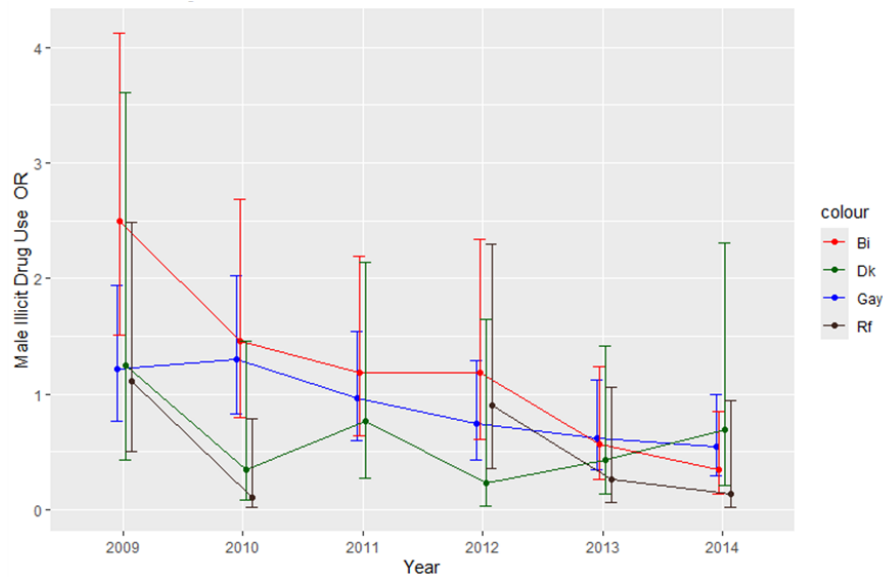

Supplement: Fig S7 — (PDF) [file pone.0305019.s007.pdf]

**Figure S8. Odds ratio disparity over time for illicit drug use for females 2009-2014**

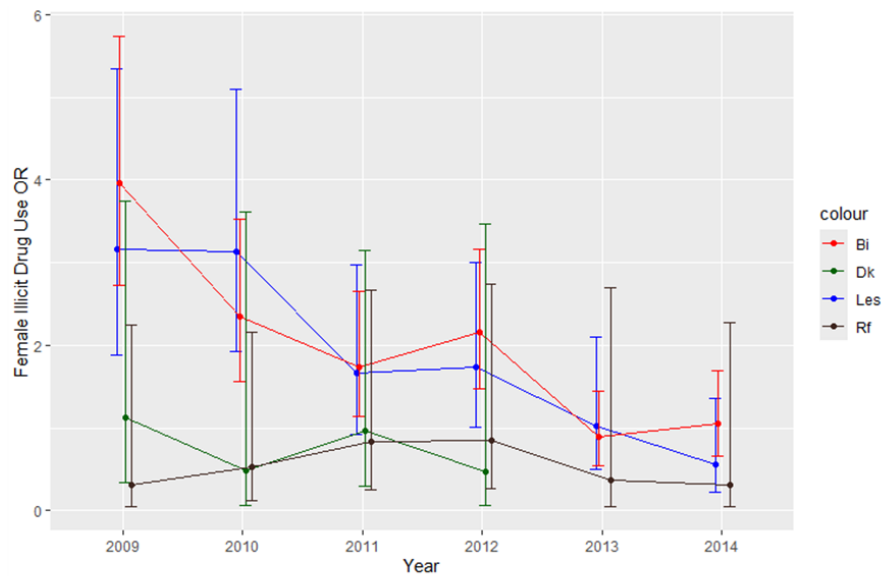

Supplement: Fig S8 — (PDF) [file pone.0305019.s008.pdf]

**Figure S9. Odds ratio disparity over time for cannabis use for males 2009-2014**

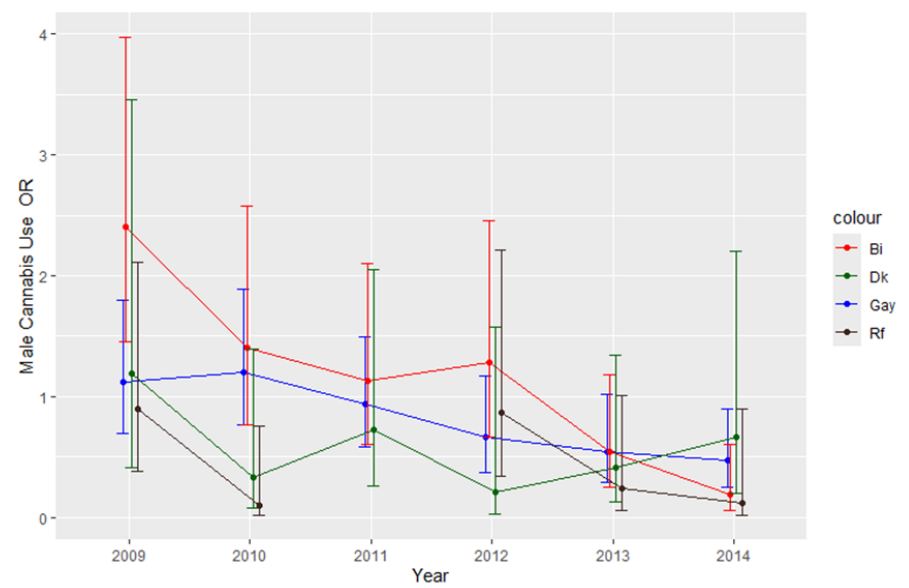

Supplement: Fig S9 — (PDF) [file pone.0305019.s009.pdf]

**Figure S10. Odds ratio disparity over time for cannabis use for females 2009-2014**

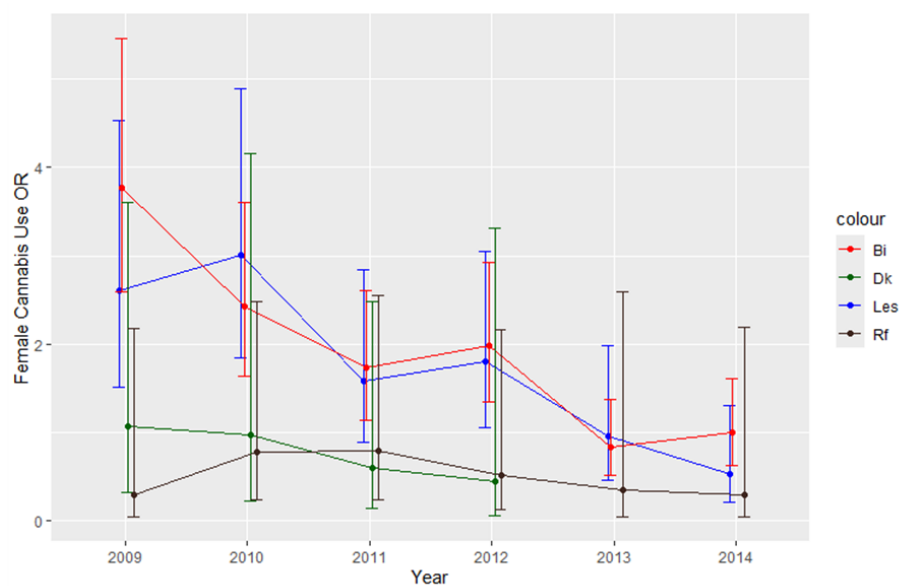

Supplement: Fig S10 — (PDF) [file pone.0305019.s010.pdf]
