# Supplementary material for: Disparities in self-reported mental health, physical health, and substance use across sexual orientations in Canada
Source: PLoS One. 2025 Mar 17;20(3):e0305019. doi: 10.1371/journal.pone.0305019 (PMC11913302; doi:10.1371/journal.pone.0305019)
Supplement: Table S1 — (PDF) [file pone.0305019.s011.pdf]

Table S1. Results for Mann-Kendall Trend Test across poor mental health, poor physical health, binge drinks, illicit drug use, and cannabis use across sex and

	Mental Health	Physical Health	Binge Drinks	Illicit Drug Use	Cannabis Use
**Gay men**	tau = 0.200<br>p = 0.707	tau = -0.333<br>p = 0.452	tau = -0.067<br>p = 1.000	tau = -0.867<br>p = 0.024	tau = -0.867<br>p = 0.024
**Bisexual men**	tau = 0.733<br>p = 0.060	tau = 0.600<br>p = 0.133	tau = -0.733<br>p = 0.060	tau = -0.867<br>p = 0.024	tau = -0.867<br>p = 0.024
**Don't Know men**	tau = -0.467<br>p = 0.260	tau = 0.733<br>p = 0.060	tau = -0.333<br>p = 0.452	tau = -0.200<br>p = 0.707	tau = -0.200<br>p = 0.707
**Refused men**	tau = -0.333<br>p = 0.452	tau = -0.200<br>p = 0.707	tau = -0.200<br>p = 0.707	tau = -0.400<br>p = 0.462	tau = -0.400<br>p = 0.462
**Lesbian**	tau = 0.333<br>p = 0.452	tau = -0.067<br>p = 1.000	tau = 0.200<br>p = 0.707	tau = -0.867<br>p = 0.024	tau = -0.733<br>p = 0.060
**Bisexual women**	tau = 0.867<br>p = 0.024	tau = -0.467<br>p = 0.260	tau = 0.200<br>p = 0.707	tau = -0.733<br>p = 0.060	tau = -0.733<br>p = 0.060
**Don't Know women**	tau = 0.333<br>p = 0.452	tau = 0.733<br>p = 0.060	tau = 0.600<br>p = 0.133	tau = -0.667<br>p = 0.308	tau = -1.000<br>p = 0.089429
**Refused women**	tau = 0.067<br>p = 1.000	tau = -0.333<br>p = 0.452	tau = 0.333<br>p = 0.452	tau = 0.067<br>p = 1.000	tau = -0.200<br>p = 0.707

sexual orientation for Canadians from 2009-2014

[Note] Data from 2009 to 2014. Model data is controlled for variables including year of birth, marital status, educational attainment, student status, self-reported ethnic minority status, employment status, rurality status, province of residence, year of interview, and federal income.
