# Supplementary material for: Disparities in self-reported mental health, physical health, and substance use across sexual orientations in Canada
Source: PLoS One. 2025 Mar 17;20(3):e0305019. doi: 10.1371/journal.pone.0305019 (PMC11913302; doi:10.1371/journal.pone.0305019)
Supplement: Table S2 — (PDF) [file pone.0305019.s012.pdf]

**Table S2. Weighted number of item non-response (including don't know and refused) for all variables**

| Variable | Item non-response, N (% of total responses) |
|---|---|
| Poor Mental Health | 19,000 (0.10%) |
| Poor Physical Health | 13,000 (0.07%) |
| Cannabis Use | 84,000 (0.42%) |
| Illicit Drug Use | 33,000 (0.17%) |
| Binge Drank Alcohol | 80,000 (0.40%) |
| Married | 29,000 (0.15%) |
| Has Completed Post-Secondary Education | 126,000 (0.63%) |
| Current Student | 0 (0.00%) |
| Ethnicity | 827,000 (4.14%) |
| Employed in Last Year | 3,000 (0.02%) |
| Lives Rural | 0 (0.00%) |
| Year of Interview | 0 (0.00%) |
| Residing Region | 0 (0.00%) |
| Income Quintile | 898,000 (4.49%) |

[Note] **Data pooled from 2009 to 2014, total weighted sample size n=19,980,000 individuals.**
