# Supplementary material for: Disparities in self-reported mental health, physical health, and substance use across sexual orientations in Canada
Source: PLoS One. 2025 Mar 17;20(3):e0305019. doi: 10.1371/journal.pone.0305019 (PMC11913302; doi:10.1371/journal.pone.0305019)
Supplement: Table S3 — (PDF) [file pone.0305019.s013.pdf]

Table S3. Male poisson risk ratios and unimputed odds ratios for poor physical health, poor mental health, binge drinking, using illicit drugs, and using cannabis across sex and sexual orientation for Canadians

Sexual Orientation	Risk Ratio (95% CI)	p-value	Odds Ratio (95% CI)	p-value
**Model**	**Poisson models**		**Unimputed models**	
	**Model 1c: Poor Mental Health (Male)**		**Model 1d: Poor Mental Health (Male)**	
**Heterosexual**	Reference		Reference	
**Homosexual**	1.48 (1.13-1.92)	0.004	1.68 (1.21-2.35)	0.002
**Bisexual**	2.20 (1.77-2.74)	0.000	2.58 (1.81-3.67)	0.000
**Don't Know**	0.97 (0.56-1.67)	0.900	0.94 (0.44-2.02)	0.871
**Refuse**	1.21 (0.64-2.30)	0.557	1.46 (0.53-4.02)	0.459
	**Model 2c: Poor Physical Health (Male)**		**Model 2d: Poor Physical Health (Male)**	
**Heterosexual**	Reference		Reference	
**Homosexual**	1.17 (0.92-1.49)	0.198	1.27 (0.92-1.75)	0.150
**Bisexual**	1.49 (1.08-1.81)	0.010	1.56 (1.05-2.31)	0.027
**Don't Know**	0.69 (0.44-1.09)	0.110	0.65 (0.33-1.27)	0.208
**Refuse**	1.13 (0.67-1.92)	0.639	1.63 (0.74-3.60)	0.223
	**Model 3c: Binge Drinks Alcohol (Male)**		**Model 3d: Binge Drinks Alcohol (Male)**	
**Heterosexual**	Reference		Reference	
**Homosexual**	0.98 (0.91-1.05)	0.557	0.88 (0.71-1.09)	0.256
**Bisexual**	0.98 (0.87-1.10)	0.710	0.97 (0.67-1.42)	0.887
**Don't Know**	0.56 (0.39-0.81)	0.002	0.27 (0.15-0.49)	0.000
**Refuse**	0.63 (0.47-0.84)	0.001	0.37 (0.21-0.64)	0.000
	**Model 4c: Uses Illicit Drugs (Male)**		**Model 4d: Uses Illicit Drugs (Male)**	
**Heterosexual**	Reference		Reference	
**Homosexual**	1.14 (0.84-1.56)	0.398	1.10 (0.73-1.65)	0.649
**Bisexual**	1.26 (0.89-1.79)	0.187	1.39 (0.85-2.28)	0.192
**Don't Know**	0.18 (0.09-0.34)	0.000	0.11 (0.04-0.28)	0.000
**Refuse**	0.46 (0.24-0.88)	0.020	0.32 (0.12-0.83)	0.019
	**Model 5c: Uses Cannabis (Male)**		**Model 5d: Uses Cannabis (Male)**	
**Heterosexual**	Reference		Reference	
**Homosexual**	1.11 (0.80-1.55)	0.525	1.13 (0.73-1.74)	0.594
**Bisexual**	1.16 (0.82-1.64)	0.397	1.24 (0.76-2.01)	0.386

**Don't Know**	0.17 (0.09-0.33)	0.000	0.10 (0.04-0.27)	0.000
**Refuse**	0.41 (0.21-0.82)	0.012	0.25 (0.09-0.71)	0.009

[Note] **Data pooled from 2009 to 2014, total weighted sample size n=19,980,000 individuals. Adjusted models are controlled for variables including year of birth, marital status, educational attainment, student status, self-reported ethnic minority status, employment status, rurality status, province of residence, year of interview, and federal income.**
