# Supplementary material for: Disparities in self-reported mental health, physical health, and substance use across sexual orientations in Canada
Source: PLoS One. 2025 Mar 17;20(3):e0305019. doi: 10.1371/journal.pone.0305019 (PMC11913302; doi:10.1371/journal.pone.0305019)
Supplement: Table S4 — (PDF) [file pone.0305019.s014.pdf]

**Table S4. Female poisson risk ratios and unimputed odds ratios for poor physical health, poor mental health, binge drinking, using illicit drugs, and using cannabis across sex and sexual orientation for Canadians**

| Sexual Orientation | Risk Ratio (95% CI) | p-value | Odds Ratio (95% CI) | p-value |
|---|---|---|---|---|
| **Model** | **Poisson models** | | **Unimputed models** | |
| | **Model 6c: Poor Mental Health (Female)** | | **Model 6d: Poor Mental Health (Female)** | |
| **Heterosexual** | Reference | | Reference | |
| **Homosexual** | 1.26 (0.96-1.65) | 0.090 | 1.33 (0.95-1.86) | 0.099 |
| **Bisexual** | 2.64 (2.21-3.17) | 0.000 | 3.47 (2.64-4.57) | 0.000 |
| **Don't Know** | 1.03 (0.63-1.68) | 0.904 | 1.18 (0.61-2.29) | 0.627 |
| **Refuse** | 1.30 (0.78-2.18) | 0.317 | 0.88 (0.41-1.90) | 0.749 |
| | **Model 7c: Poor Physical Health (Female)** | | **Model 7d: Poor Physical Health (Female)** | |
| **Heterosexual** | Reference | | Reference | |
| **Homosexual** | 1.13 (0.87-1.46) | 0.369 | 1.22 (0.86-1.73) | 0.267 |
| **Bisexual** | 2.00 (1.65-2.41) | 0.000 | 2.53 (1.89-3.40) | 0.000 |
| **Don't Know** | 0.87 (0.56-1.36) | 0.532 | 0.69 (0.36-1.33) | 0.266 |
| **Refuse** | 1.16 (0.71-1.89) | 0.563 | 1.35 (0.58-3.16) | 0.492 |
| | **Model 8c: Binge Drinks Alcohol (Female)** | | **Model 8d: Binge Drinks Alcohol (Female)** | |
| **Heterosexual** | Reference | | Reference | |
| **Homosexual** | 1.20 (1.08-1.33) | 0.001 | 1.53 (1.14-2.05) | 0.005 |
| **Bisexual** | 1.21 (1.13-1.30) | 0.000 | 1.61 (1.28-2.03) | 0.000 |
| **Don't Know** | 0.50 (0.35-0.70) | 0.000 | 0.33 (0.21-0.54) | 0.000 |
| **Refuse** | 0.67 (0.45-1.00) | 0.050 | 0.58 (0.26-1.32) | 0.196 |
| | **Model 9c: Uses Illicit Drugs (Female)** | | **Model 9d: Uses Illicit Drugs (Female)** | |
| **Heterosexual** | Reference | | Reference | |
| **Homosexual** | 1.74 (1.27-2.38) | 0.001 | 1.85 (1.26-2.71) | 0.002 |
| **Bisexual** | 2.08 (1.61-2.67) | 0.000 | 2.32 (1.62-3.30) | 0.000 |
| **Don't Know** | 0.30 (0.09-0.99) | 0.047 | 0.22 (0.04-1.17) | 0.076 |
| **Refuse** | 0.69 (0.21-2.21) | 0.526 | 0.48 (0.14-1.67) | 0.249 |
| | **Model 10c: Uses Cannabis (Female)** | | **Model 10d: Uses Cannabis (Female)** | |
| **Heterosexual** | Reference | | Reference | |

| | | | | |
|---|---|---|---|---|
| **Homosexual** | 1.68 (1.22-2.31) | 0.002 | 1.79 (1.21-2.64) | 0.003 |
| **Bisexual** | 2.07 (1.62-2.65) | 0.000 | 2.36 (1.66-3.34) | 0.000 |
| **Don't Know** | 0.36 (0.13-1.03) | 0.056 | 0.31 (0.08-1.16) | 0.081 |
| **Refuse** | 0.66 (0.21-2.09) | 0.476 | 0.45 (0.12-1.62) | 0.220 |

[Note] **Data pooled from 2009 to 2014, total weighted sample size n=19,980,000 individuals. Adjusted models are controlled for variables including year of birth, marital status, educational attainment, student status, self-reported ethnic minority status, employment status, rurality status, province of residence, year of interview, and federal income.**
