# Supplementary material for: Disparities in self-reported mental health, physical health, and substance use across sexual orientations in Canada
Source: PLoS One. 2025 Mar 17;20(3):e0305019. doi: 10.1371/journal.pone.0305019 (PMC11913302; doi:10.1371/journal.pone.0305019)
Supplement: Table S5 — (PDF) [file pone.0305019.s015.pdf]

Table S5. Second-difference test to estimate the difference of (1) - (2): (1) difference between urban sexual minority groups vs urban heterosexual individuals and (2) difference between rural sexual minority groups vs rural heterosexual individuals. Sexual minority groups include gay/lesbian, bisexual, and Don't know/refuse.

Mental Health	Men		Women	
	Predicted probabilities (standard errors)	p-value	Predicted probabilities (standard errors)	p-value
Difference in Gay/Lesbian vs. Heterosexual individuals in urban vs. rural	0.086 (0.065)	0.184	0.010 (0.033)	0.756
Difference in Bisexual vs. Heterosexual individuals in urban vs. rural	-0.043 (0.033)	0.183	-0.052 (0.040)	0.189
Difference in Don't Know/Refuse vs. Heterosexual individuals in urban vs. rural	-0.009 (0.332)	0.772	-0.015 (0.029)	0.609

Physical Health	Men		Women	
	Predicted probabilities (standard errors)	p-value	Predicted probabilities (standard errors)	p-value
Difference in Gay/Lesbian vs. Heterosexual individuals in urban vs. rural	0.072 (0.075)	0.340	0.036 (0.037)	0.324
Difference in Bisexual vs. Heterosexual individuals in urban vs. rural	-0.042 (0.034)	0.217	0.066 (0.049)	0.175
Difference in Don't Know/Refuse vs. Heterosexual individuals in urban vs. rural	-0.066 (0.032)	0.040	-0.025 (0.034)	0.454

Substance use	Men		Women	
	Predicted probabilities (standard errors)	p-value	Predicted probabilities (standard errors)	p-value
Difference in Gay/Lesbian vs. Heterosexual individuals in urban vs. rural	-0.079 (0.072)	0.276	0.156 (0.082)	0.060
Difference in Bisexual vs. Heterosexual individuals in urban vs. rural	0.064 (0.076)	0.403	-0.069 (0.06)	0.251
Difference in Don't Know/Refuse vs. Heterosexual individuals in urban vs. rural	-0.004 (0.105)	0.968	-0.113 (0.08)	0.159
